# Activity fingerprinting of AMR β-lactamase towards a fast and accurate diagnosis

**DOI:** 10.3389/fcimb.2023.1222156

**Published:** 2023-09-05

**Authors:** Chenchen Song, Xuan Sun, Yao Wang, Leif Bülow, Michael Mecklenburg, Changxin Wu, Qinglai Meng, Bin Xie

**Affiliations:** ^1^ Institute of Biomedical Sciences, The Key Laboratory of Chemical Biology and Molecular Engineering of National Ministry of Education, Shanxi Provincial Key Laboratory of Medical Molecular Cell Biology, Shanxi University, Taiyuan, China; ^2^ Division of Pure and Applied Biochemistry, Department of Chemistry, Lund University, Lund, Sweden; ^3^ School of Basic Medical Sciences, Hubei University of Chinese Medicine, Wuhan, China; ^4^ Omik Bioscience AB, Lund, Sweden

**Keywords:** antibiotics, antimicrobial resistance, NDM-1, thermometric biosensor, β-lactamase

## Abstract

Antibiotic resistance has become a serious threat to global public health and economic development. Rapid and accurate identification of a patient status for antimicrobial resistance (AMR) are urgently needed in clinical diagnosis. Here we describe the development of an assay method for activity fingerprinting of AMR β-lactamases using panels of 7 β-lactam antibiotics in 35 min. New Deli Metallo β-lactamase-1 (NDM-1) and penicillinase were demonstrated as two different classes of β-lactamases. The panel consisted of three classes of antibiotics, including: penicillins (penicillin G, piperacillin), cephalosporins (cefepime, ceftriaxone, cefazolin) and carbapenems (meropenem and imipenem). The assay employed a scheme combines the catalytic reaction of AMR β-lactamases on antibiotic substrates with a flow-injected thermometric biosensor that allows the direct detection of the heat generated from the enzymatic catalysis, and eliminates the need for custom substrates and multiple detection schemes. In order to differentiate classes of β-lactamases, characterization of the enzyme activity under different catalytic condition, such as, buffer composition, ion strength and pH were investigated. This assay could provide a tool for fast diagnosis of patient AMR status which makes possible for the future accurate treatment with selected antibiotics.

## Introduction

Since the discovery of penicillin, a broad range of antibiotics have been discovered which have enhanced our ability to treat bacterial infections. Antibiotics have played a critical role in medicine, as well as in other fields (Bennett and Chung, 2001). However, in recent years, the dramatic increase in use of antibiotics has resulted in the spread of antibiotic resistance. Initially, resistance was limited to a single antibiotic and was easily addressed by simply choosing another antibiotic. As resistance has spread, a class of pathogens emerged that are resistant to multiple antibiotics, so-called multi-resistant pathogens. This trend has continued and spawned a new class of pathogens which are resistant to all known antibiotics, i.e. super-bugs. Patients infected with these pathogens have dramatically higher hospitalization and mortality rates (Hansen et al., 2015). This trend in AMR threatens to undermine global public health and economic development (English and Gaur, 2010; Qiao et al., 2018).

Physicians are on the front lines in the effort to treat patients infected with multi-resistant pathogens. However, in order to determine the most effective course of treatment, physicians require AMR diagnostics capable of rapidly and specifically determining the patient’s AMR profile. Although there are multiple mechanisms by which pathogens achieve resistance, the most common mechanism is the production of enzymes that catalytically inactivate the antibiotics. Numerous physical and activity based methods have been developed to detect these AMR enzymes ranging from traditional methods, such as the disk method to new techniques, such as multiplex PCR, DNA microarrays, colorimetric assays, immune-chromatographic technique and thermometric analysis. Each technique has advantages and disadvantages. However, none of these techniques is capable of providing results/diagnostics of multi-resistance in a clinically relevant timeframe. Efforts must be renewed to address this issue (Rodriguez-Rojas et al., 2013; Blair et al., 2014). Rapid, simple, and reliable AMR analytical solution are urgently needed that provide doctors with the ability to profile AMR in order to allow tailored, personalized therapeutics for patients infected with AMR pathogens.

There are several methods that have been conventionally used to detect antibiotic resistance in recent decades, such as the double dilution method and susceptibility testing (Ge et al., 2013). However, these traditional detection methods have low sensitivity, are complicated process and time-consuming. Thus, researchers have exploited some other methods, including multiplex PCR, immune-chromatographic techniques, colorimetric methods, DNA microarray, and thermometric biosensor (March-Rosselló, 2017). Of all the above methods, the thermometric method has attracted our attention because of its rapid analysis, simple operation, and high throughput (Chen et al., 2015; Wang et al., 2018).

Here we describe the development of a novel AMR strategy that profiles AMR enzymes by testing a panel of antibiotics. The diagnostic strategy specifically and efficiently identifies the AMR enzymatic activity profile using a flow injected thermal biosensor device. For these studies, we have chosen to use a panel of antibiotics that are widely used in clinical settings. The panel consisted of three different classes of antibiotics: Penicillins (penicillin G, piperacillin), Cephalosprins (cefazolin, ceftriaxone, cefepime) and Carbapenems (imipenem, meropenem). In order to verify the profile based characterization, two classes of AMR β-lactamases: New Deli Metallo β-lactamase-1 (NDM-1) and penicillinase were chosen as examples in the study. The effects of the reaction conditions, such as: ion strength, buffer composition, and pH on the activity profiling of the AMR β-lactamases catalyzing this panel of antibiotics were investigated.

## Materials and methods

### Chemicals and materials

Penicillin G, Piperacillin, Ceftriaxone, Cefepime, Cefazolin, Meropenem were purchased from Solarbio Co., Ltd. (Beijing, China). Imipenem was bought from Fresenius Kabi (Bad Homburg, Germany). HEPES was obtained from Sangon Biotech Co., Ltd. (Shanghai, China). All other reagents were purchased from Sigma-Aldrich Co (St. Louis, USA). The controlled pore glass (CPG) beads (125–140 μm particle diameter and 50 nm pore size) were originally purchased from VEB Trisola, Steinach, Germany. The construction of plasmid encoding NDM-1, expression and purification of recombinant NDM-1(rNDM-1) were performed as previously described (Meng et al., 2021a; Meng et al., 2021b). The purity of the purified rNDM-1 is > 95%, The unit activity of rNDM-1 to hydrolyze meropenem is 108 IU/mg.

### Buffer and sample preparation

Phosphate buffer (PB) and HEPES buffer in different ionic strengths (0.01, 0.05 0.1 M) and pHs (pH 6.8, 7.2, 7.6, 8.0) were prepared as running buffers and for sample preparation. All water used for preparing the buffers and samples was from a Milli-Q system (Millipore, Bedford, MA, USA).

Stock solutions of each of the antibiotics: Penicillin G, Piperacillin, Ceftriaxone, Cefepime, Cefazolin, Meropenem, and Imipenem, were prepared in PB and HEPES at the various ionic strengths and pHs, which were then serially diluted: 10 mM, 5 mM, 2.5 mM, 1.25 mM, and 0.625 mM.

### The analytical strategy

The Enzyme Thermal biosensor (ET) is a flow-injected analytical device (Omik Bioscience AB, Sweden) has been employed in this study. The enzyme, e.g. β-lactamase, was immobilized on spherical porous glass beads (CPG), loaded in an enzyme column and substrate samples were injected (e.g. antibiotics) with running buffers at various concentrations. Meanwhile the simultaneously generated heat can be precisely and continuously detected with high resolution thermistor sensors (Ramanathan and Danielsson, 2001; Zhou et al., 2013). The thermistor sensor converts the heat generated by the reaction into a temperature signal (Xie et al., 2000; Qie et al., 2013). In an adiabatic system, if all the heat released is used to increase the temperature of the reaction system, the following formula can be used:


$$\Delta T=-\frac{n\Delta H}{C_{S}}$$

Where *n* is the number of moles of product; Δ*H* is the enthalpy change of the biochemical reaction; Δ*T* is the temperature change of the reaction system; *C_S_* is the heat capacity of the reaction system. This formula shows that there is a linear relationship between the temperature change and the substrate concentration. Thus, the peak height or area can be used for determination of an analyte concentration (Xie et al., 2000; Yakovleva et al., 2013). Thermometric biosensors have numerous advantages including: the universality of the sensing mechanism, label-free analysis and high specificity. In addition, thermal sensors are less sensitive to sample interference than optical and electrical biosensors. The technology has been widely used to detect sample concentration and enzyme activity in clinical analysis, fermentation process control, food safety and environmental monitoring (Xie et al., 1994; Yakovleva et al., 2012; Chen et al., 2013; Yao et al., 2015; Andersson et al., 2017; Xu et al., 2017; Adlerberth et al., 2020).

**Figure 1:**
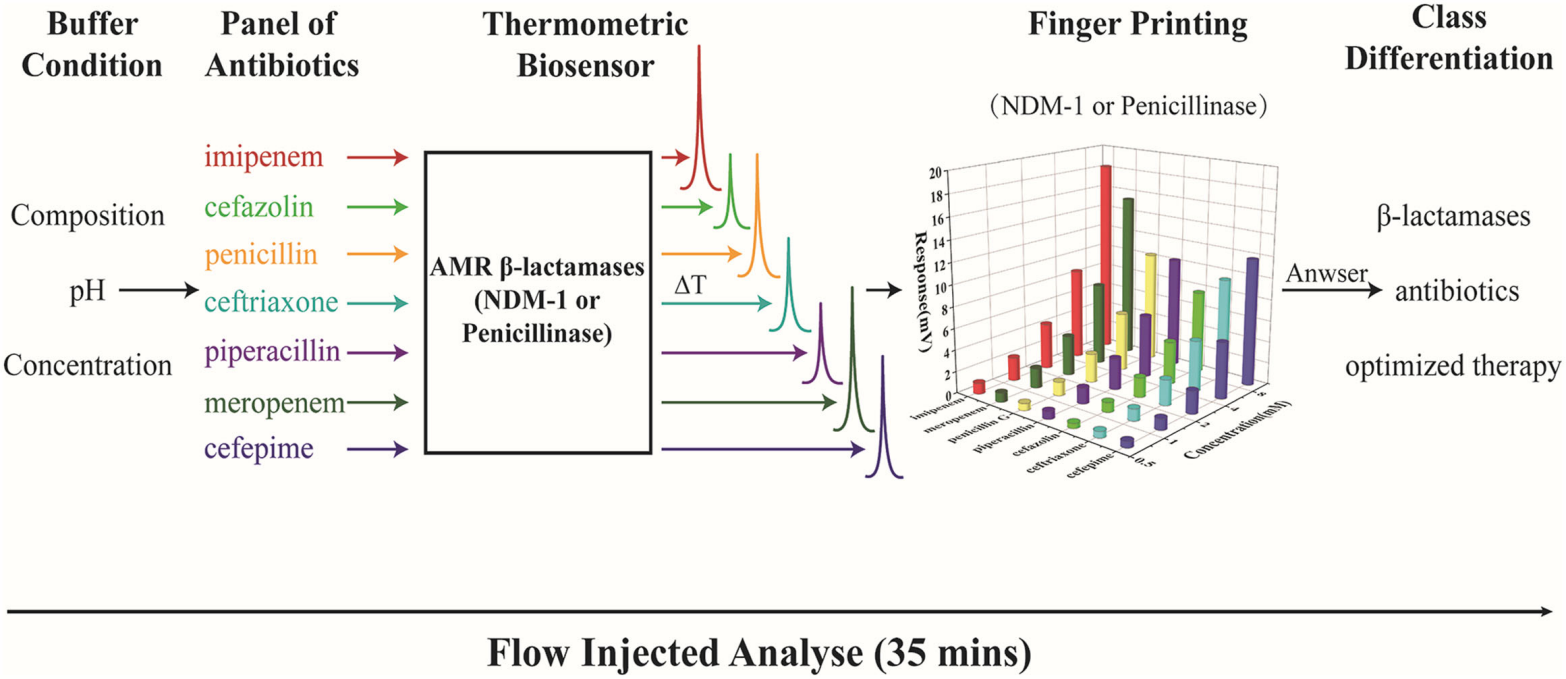
Diagram for fast classification of AMR β-lactamases using a thermometric biosensor profiling of the catalytic activity.

Here, we establish a novel method for fast identification and classification of AMR β-lactamases by thermometric biosensor profiling of the enzyme catalytic activity for a panel of antibiotics (**Figure 1**). The device is simple to operate and automatically monitors the degradation reactions of various antibiotics. Considering the clinical interesting, 7 mostly used antibiotics including penicillin G, piperacillin, cefepime, ceftriaxone, cefazolin, meropenem, and imipenem were selected for the NDM-1 and penicillinase profiling studies.

### Instrumental setup

The Enzyme Thermal biosensor used in this study was composed of a pump, a sample valve, split two enzyme columns (one as measurement and the other as reference) and high resolution thermistors in coupled with signal amplification.

The enzyme column was loaded with ca. 50 IU immobilized β-lactamase (e.g. penicillinase or NDM-1) onto CPG. The working temperature was set at 25°C. A flow rate of 0.6 mL min^-1^ and sample volumes of 30 and 75 μL were used. However, for the comparative study, the response signals were adjusted to 10 μL/analyte. Each sample measurement was performed in triplicate.

## Results

### Effects of buffer composition and concentration on profiling of the AMR β-lactamases

We first evaluated effects of different buffers on degradation of 7 β-lactam antibiotics: penicillin G, piperacillin, ceftriaxone, cefepime, cefazolin, meropenem and imipenem. Among these 7 antibiotics, penicillin G and piperacillin belong to penicillin class; ceftriaxone, cefepime, cefazolin belong to cephalosporin class; and meropenem and imipenem belong to carbapenem class. Cefazolin, ceftriaxone and cefepime are representative of the 1^st^, 3^rd^ and 4^th^ generation cephalosporins, respectively.

**Figure 2:**
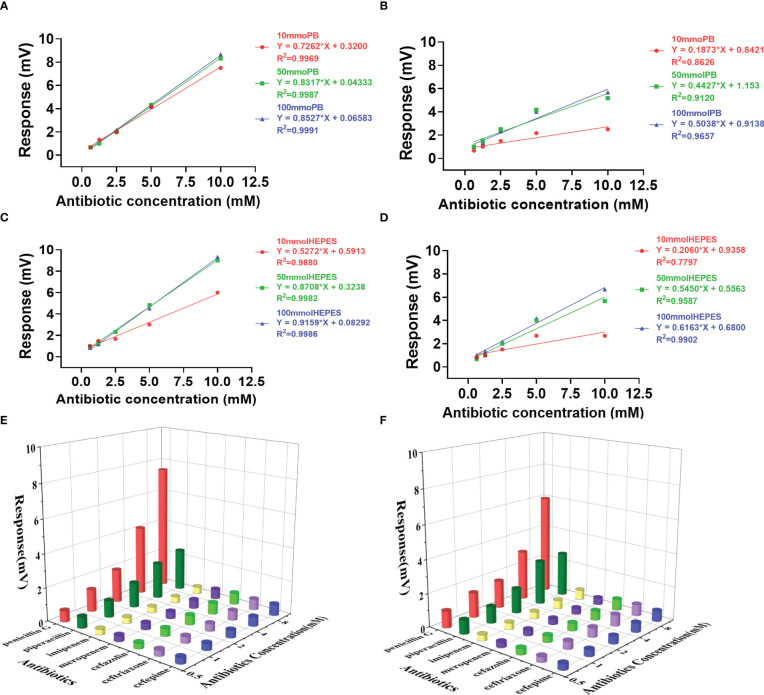
Effects of buffer on the activity and profiling of penicillinase catalysis. Response slopes in different concentrations of Penicillin G and Piperacillin in PB buffer (**A, B**, respectively) and HEPES buffer (**C, D**, respectively). The profile generated with a panel of 7 antibiotics in PB buffer **(E)** and HEPES buffer **(F)** using 10 mM concentration.

**Figure 3:**
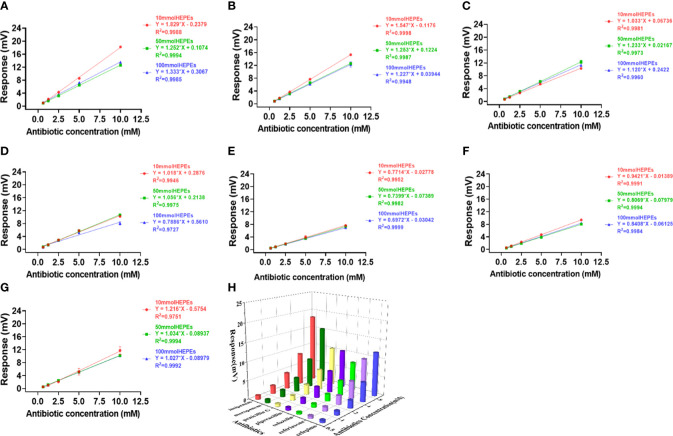
Effects of HEPES buffer on the activity and profiling of NDM-1 catalysis. The response slopes of 7 antibiotics in buffer concentrations of 10 mM (red), 50 mM (green) and 100 mM (blue) were presented by **(A)** Imipenem, **(B)** Meropenem, **(C)** Penicillin G, **(D)** Piperacillin, **(E)** Cefazolin, **(F)** Ceftriaxone, **(G)** Cefepime. The catalytic profile was generated using a 10 mM PB buffer **(H)**.

Studies were performed to determine the effect that buffer had on assay sensitivity (**Figures 2**, **3**). In this study, thermometric biosensor was employed for instant detection of the catalytic enthalpy (e.g. NDM-1 or penicillinase catalysis of antibiotics) that was converted to a corresponding temperature changes. The intensity of the thermal signal was proportional to the concentration of the substrates, e.g. antibiotics. In this study, a panel of 7 different antibiotics at concentrations of 0.625, 1.25, 2.5, 5.0 and 10 mM were determined sequentially. Therefore, a complete profile for one AMR enzyme could be generated in 35 min based on a cycle time of 5 min.

### Penicillinase profiling

As penicillinase can only efficiently catalyze penicillin class, only penicillin G and piperacillin response curves were presented in buffers of PB (**Figures 2A, B**) and HEPES (**Figures 2C, D**) at a concentration of 10, 50 and 100 mM. However, the 3D profiles of the catalytic activities for the 7 antibiotics were generated using a concentration of 10 mM using PB and HEPES buffers (**Figures 2E, F**).

The two buffer systems presented similar sensitivities for the individual antibiotics. However, when comparing the three buffer concentrations, the sensitivity was the highest at 100 mM and lowest at 10 mM. Meanwhile the highest variation in sensitivity was seen at 10 mM buffer concentration for both PB and HEPES (**Figure 2**). The lowest sensitivity variation was observed for Penicillin G in PB, which means penicillinase catalyzing penicillin G was relatively stable in PB buffer.

### NDM-1 profiling

**Figure 4:**
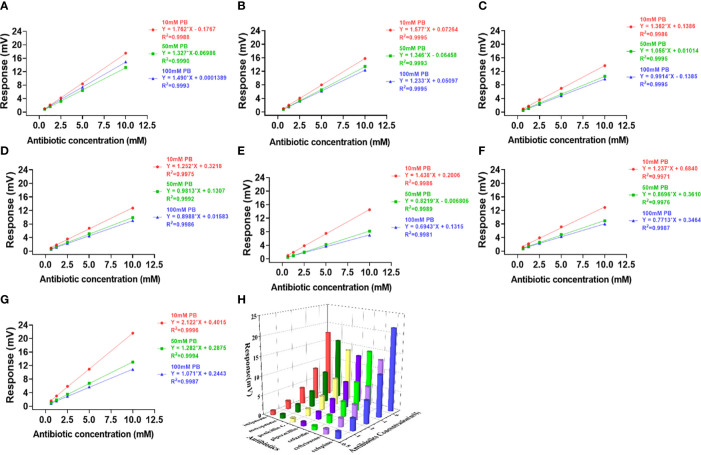
Effects of PB buffer on the activity and profiling of NDM-1 catalysis. The response slopes of 7 antibiotics in buffer concentrations of 10 mM (red), 50 mM (green) and 100 mM (blue) were presented by **(A)** Imipenem, **(B)** Meropenem, **(C)** Penicillin G, **(D)** Piperacillin, **(E)** Cefazolin, **(F)** Ceftriaxone, **(G)** Cefepime. The catalytic profile was generated using 10 mM PB buffer **(H)**.

In order to verify the catalytic efficiency of NDM-1 enzyme, three classes of antibiotics including: Penicillins (penicillin G, piperacillin), cephalosporins (cefepime, ceftriaxone, cefazolin) and Carbapenems (meropenem, imipenem), were investigated using PB (**Figure 4**) and HEPES (**Figure 3**) buffers at concentrations of 10, 50 and 100 mM.

In contrast to Penicillinase in **Figure 2**, the highest sensitivity was obtained with 10 mM buffer and the lowest with 100 mM buffer for both PB (**Figure 4**) and HEPES (**Figure 3**) for all 7 antibiotics with the exception of penicillin G in HEPES buffer (**Figure 3C**). In addition, effect of the buffer concentration on profile variation was the highest in PB (**Figures 4E-G**), but the lowest in HEPES buffer (**Figures 3E-G**) for the Cephalosprins antibiotic class (cefepime, ceftriaxone, cefazolin). This finding could be possibly used for differentiating cephalosprins from other classes of antibiotics.

**Figure 5:**
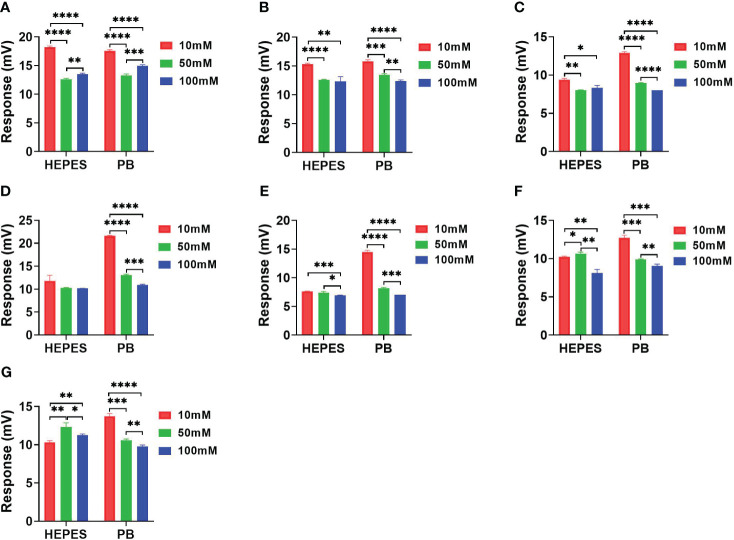
The response comparison between PB and HEPES buffers in 10, 50 and 100 mM concentrations under NDM-1 catalyzation of 7 antibiotics. **(A)** Imipenem, **(B)** Meropenem, **(C)** Penicillin G, **(D)** Piperacillin, **(E)** Cefazolin, **(F)** Ceftriaxone, **(G)** Cefepime. P value: * < 0.05, ** < 0.01, *** < 0.001, **** < 0.0001.

**Figure 5** showing the signal response comparison between PB and HEPES buffers was similar to those seen in **Figure 3** and **4**, i.e. that the highest sensitivity was obtained at 10 mM buffer for almost all antibiotic reactions. Cefazolin showed the lowest response indicating that it generated the least catalytic enthalpy as compared with the other antibiotics. In general, higher sensitivity was observed in PB than HEPES buffer.

### pH effects on the profiling

**Figure 6:**
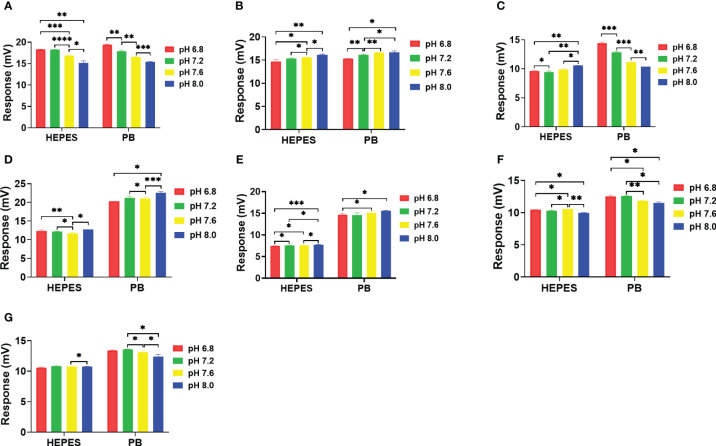
Effect of pH on NDM-1catalysis of 7 antibiotics in 10 mM. **(A)** Imipenem, **(B)** Meropenem, **(C)** Penicillin G, **(D)** Piperacillin, **(E)** Cefazolin, **(F)** Ceftriaxone, **(G)** Cefepime. P value: * < 0.05, ** < 0.01, *** < 0.001, **** < 0.0001.

The pH effects on the catalytic reactions were evaluated in PB and HEPES buffers at pHs 6.8, 7.2, 7.6 and 8.0 (**Figure 6**). Using the HEPES buffer, an apparent pH dependent effect on catalytic activity of NDM-1 was observed in both carbapenem antibiotics (**Figures 6A, B**). In addition, an apparent pH dependent effect on catalytic activity of NDM-1 was observed in both carbapenem and penicillin antibiotics using PB buffer (**Figures 6A-D**). In addition, the results show that higher sensitivity was observed using PB than HEPES, and the sensitivity variation was also higher for PB than HEPES. Meanwhile, the cefazolin showed the smallest response in HEPES buffer and was least effected by pH. Overall, the differential effect of pH on the catalytic activity of NDM-1 and penicillinase might facilitate differentiation of these two β-lactamases.

## Discussion

As enzymatic degradation of antibiotics is the most common mechanism of AMR, we have developed a thermometric biosensor strategy for the rapid identification of AMR enzymes in 35 min using a panel of antibiotics to profile their substrate specificity. In this study, a substrate specificity profile for NDM-1 and penicillinase has been created using a panel of 7 antibiotics including penicillin G, piperacillin, cefepime, ceftriaxone, cefazolin, meropenem, and imipenem. The results show that the strategy is both feasible and reliable.

The concentration dependent calibration curves were prepared using PB and HEPES. The results show that buffer conditions can be employed to further increase the informational content of the assay scheme should the antibiotic profile alone be inadequate to identify the AMR enzyme. The results of the buffer and pH effects on the profiles provided the possibility for differentiation of classes of antibiotics, e.g. carbapenem and peniciilin or cephalosporin in which the carbapenem antibiotics showed highest sensitivity vs cepalosprin which showed the smallest changes in buffer concentration of 100 mM. This conclusion is also supported by the pH studies. Furthermore, the results also indicate that it is feasible to differentiate β-lactamases, e.g. penicillinase and NDM-1 by varying the buffer concentrations. The lowest buffer concentration (10 mM) gave the highest sensitivity for penicillinase reactions vs the lowest sensitivity for NDM-1 reactions but the highest sensitivity in 100 mM buffer.

As expected, penicillinase was most active against the penicillin class of antibiotics, penicillin and Piperacillin. In contrast, NDM-1 could hydrolize all three classes of antibiotics in particular carbapenems. Compared to other methods (see **Supplement Table 1**), our strategy has a number of distinct advantages. First, the flow injected assays are cost effective and simple to automate. Second, the immobilized β-lactamases were very stable and could be repeatedly used for hundreds of samples which reduces recalibration requirements. Third, substrate specificity profiles for other AMR enzymes can easily be obtained by simply exchanging the enzyme column and repeating the analysis of the panel of antibiotics. Identification of very related AMR enzymes may require the antibiotic panel to be modified. This is a simple to implement since no specialized substrates or assay modifications are needed. By expanding the β-lactamase activity profiling database, the assay will not only be able to identify known AMR enzymes, but could, in the future also be used to identify novel AMR enzymes.

## Data availability statement

The raw data supporting the conclusions of this article will be made available by the authors, without undue reservation.

## Author contributions

CS: Investigation, comparative study, data curation, formal analysis, visualization, writing - review & editing, XS: Data curation, formal analysis, investigation, methodology, validation, visualization, writing - original draft, writing - review & editing. YW, Investigation, data curation, formal analysis, LB: Funding acquisition, review & editing, supervision, resources. MM: Review &editing, English proofreading. CW: funding acquisition, supervision QM: project administration, formal analysis, writing - review & editing, funding acquisition, resources. BX: Conceptualization, project administration, data curation, formal analysis, methodology, writing - review & editing, funding acquisition, resources, supervision. All authors contributed to the article and approved the submitted version.

## Supplementary material

Supplementary Figure 1 Reproducibility and life-time of the assay.

Supplementary Table 1 For comparison of different technologies.
