# Supplementary figures and images for: Activity fingerprinting of AMR β-lactamase towards a fast and accurate diagnosis
Source: Front Cell Infect Microbiol. 2023 Sep 5;13:1222156. doi: 10.3389/fcimb.2023.1222156 (PMC10512244; doi:10.3389/fcimb.2023.1222156)

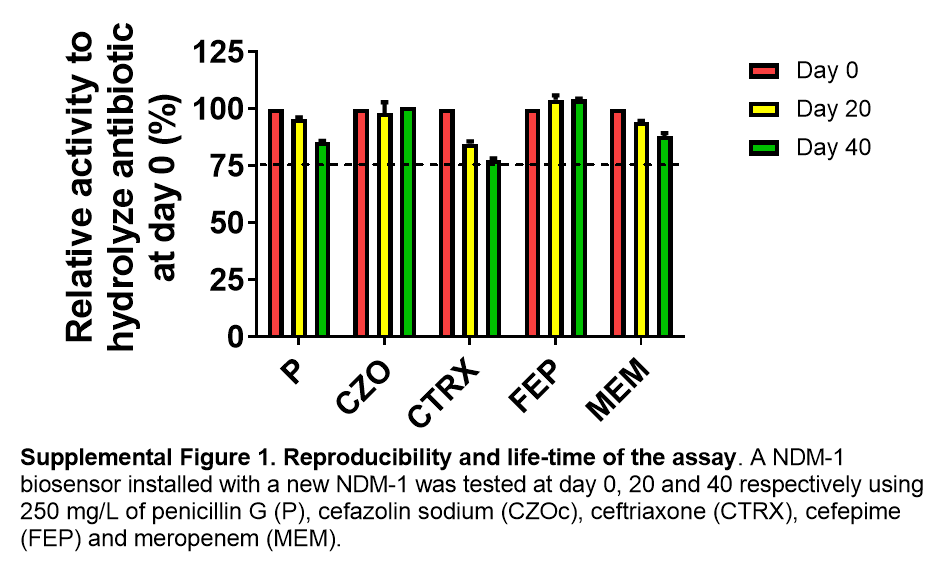

Supplement: Supplementary Figure 1 — Reproducibility and life-time of the assay. [file Image_1.tif]
