# Supplementary material for: Activity fingerprinting of AMR β-lactamase towards a fast and accurate diagnosis
Source: Front Cell Infect Microbiol. 2023 Sep 5;13:1222156. doi: 10.3389/fcimb.2023.1222156 (PMC10512244; doi:10.3389/fcimb.2023.1222156)
Supplement: Supplementary Table 1 — For comparison of different technologies. [file DataSheet_1.pdf]

Comparison of different technologies

Method	Principle	Culture/ incubation (bacteria)	Time (hour)	Multi-drug resistance	Emerging resistance	Profiling Antibiotic resistance	Susceptibility test	Point-of-care	Blood sample
**PCR**<br>*Luminex Co.,*<br>*BioFire Diagnostics,*<br>*Cepheid,*<br>*bioMérieux,*<br>*Amplex Biosystems,*<br>*OptiGene,*<br>*Amplex,*<br>*Autoimmun Diagnostika,*<br>*Roche Diagnostics,*<br>*Check-Points,*<br>*DayZero Diagnostics*<br>*DNAe Group Holdings Ltd*<br>*Genetic Signatures*<br>*Mobidiag Ltd*	Genotypic	-	>2-12	+	+	-	-	+	-
**Microarray**<br>*Check-Points*<br>*GenMark Diagnostics*<br>*InSilixa Inc.*<br>*Nanopore diagnostics*	Genotypic	-	>8	+	+	-	-	+	-
**Disc difussion**<br>*MicroScan,*<br>*bioMérieux,*<br>*BD*	Phenotypic	+	>24	+	-	+	+	-	-
**Immuno-chromatographic**<br>*Coris BioConcept*<br>*Abbott*<br>*ArcDia International Oy Ltd*	Phenotypic	+	>18	+	-	-	+	+	-
**Colorimetric**<br>*bioMérieux,*<br>*Rosco Diagnostica A/S*	Phenotypic (β-lactamases)	+	>2-6	-	-	-	+	-	-
**Imaging**<br>*Accelerate Diagnostics*<br>*Klaris*<br>*Specific Technologies*<br>*Velox Biosystem*	Phenotypic	-/+	>1-5	+	-	-	+	-	-/+
**Nephelometry**<br>*Alifax*	Phenotypic	+	>5	-	-	-	+	-	+
**MALDI-TOF**	Phenotypic	+	>3-5	-	-	+	+	-	+
**Flow cytometry**<br>*Sysmex Corp.*<br>*BacterioScan*	Phenotypic	+	>4	-	-	-	+	-	-
**Chemiluminescence /bioluminescence**	Phenotypic	+	>2-8	-	-	-	+	+	-
**Microfluidic**<br>*Astrego Diagnostics*<br>*Affinity Biosensors*<br>*GeneFluidics*	Phenotypic	+	>2-3.5	-	-	-	+	+	-
**Bacterial lysis**<br>*Avails Medical*<br>*T2 Biosystems Inc*	Genotypic	+	>5	-	-	-	+	-	-
**Calorimetry (non-flow)**<br>*SymCel Sverige AB*	Phenotypic (bacteria)	+	>5	+	-	+	+	-	-
**Flow-Thermometric Biosensor** *(Our solution)*	Phenotypic (β-lactamases)	-	<0.5-1	+	+	+	+	+	+
